# Idiopathic multicentric Castleman disease resembling autoimmune diseases: A case report

**DOI:** 10.1097/MD.0000000000043320

**Published:** 2025-07-18

**Authors:** Tongguan Li, Mengjiao Yao, Yanfeng Hou

**Affiliations:** a The First Affiliated Hospital of Shandong First Medical University & Shandong Provincial Qianfoshan Hospital, Jinan, Shandong Province, China; b Department of Rheumatology, The First Affiliated Hospital of Shandong First Medical University & Shandong Provincial Qianfoshan Hospital, Jinan, Shandong Province, China; c Shandong Province University Clinical Immunology Translational Medicine Laboratory, Jinan, Shandong Province, China.

**Keywords:** idiopathic multicentric Castleman disease, lymphadenectasis, skin erythema

## Abstract

**Rationale:**

Castleman disease (CD) is a rare lymphoproliferative disorder characterized by nonmalignant lymph node enlargement, often associated with systemic symptoms. It is classified into unicentric disease (involving a single enlarged lymph node) and multicentric disease (affecting multiple lymph node stations). In some cases of idiopathic multicentric Castleman disease (iMCD), elevated levels of various serum inflammatory markers are observed, and histologically, the lymph node enlargement resembles that caused by autoimmune diseases, making diagnosis challenging.

**Patient concerns:**

A 35-year-old female patient presented with fatigue, low-grade fever, shoulder erythema, and generalized lymphadenopathy for 2 years. Persistent systemic inflammation, anemia, thrombocytosis, hypoalbuminemia, and hyperglobulinemia were noted.

**Diagnoses:**

Blood tests revealed systemic inflammation, including elevated levels of C-reactive protein and interleukin-6, along with increased rheumatoid factor levels. Computed tomography scans showed a large, well-defined mass with uniform enhancement in the left neck. Skin erythema pathology was suggestive of allergic purpura. Bone marrow biopsy showed increased plasma cells. Lymph node pathology revealed an increase in IgG4-positive cells, with a high number of CD38 and CD138 plasma cells, and the morphology was consistent with Castleman disease (plasmacytic type). The diagnosis was iMCD.

**Interventions:**

Intravenous tocilizumab (400 mg every 4 weeks), methylprednisolone (40 mg daily), and oral thalidomide (75 mg daily) were administered. Symptomatic treatment included intravenous albumin (10 g daily), topical application of denaseide cream (0.5 g twice daily) for rash and pruritus, oral cetirizine (10 mg daily), and oral ebastine (10 mg daily).

**Outcomes:**

The patient no longer experienced low-grade fever or fatigue but continued to have lymphadenopathy and shoulder erythema.

**Lessons:**

This case highlights the rarity and uniqueness of iMCD, which can easily be confused with lymphadenopathy caused by autoimmune diseases (e.g., rheumatoid arthritis-related lymphadenopathy, IgG4-related diseases). Clinicians should consider lymph node histology in conjunction with clinical and serological findings, as well as imaging results, for accurate diagnosis.

## 1. Introduction

Castleman disease (CD) is an extremely rare lymphoproliferative disorder characterized by nonmalignant lymph node enlargement, first described by Benjamin Castleman in 1954.^[1]^ Based on the number of regions involved in the enlarged lymph nodes according to histopathological features, CD is classified into unicentric CD and multicentric CD.^[2]^ There are 3 pathological subtypes: hyaline-vascular, plasmacytic, and mixed. Multicentric Castleman disease (MCD) is typically plasmacytic or mixed type and is a systemic disease with clinical manifestations including fever, night sweats, weight loss, and fatigue.^[3]^ MCD can be classified into human herpesvirus 8-associated MCD and idiopathic MCD (iMCD).^[4]^ The pathogenesis of iMCD is not yet fully understood, but signaling pathways such as interleukin-6 (IL-6) and T cell activation may be involved.^[5]^ The prognosis of iMCD varies depending on the severity of the individual’s condition and differences in medication.

The clinical presentation, laboratory tests, and imaging findings of iMCD are similar to those of diseases such as rheumatoid arthritis (RA) and IgG4-related disease (IgG4-RD), and it is associated with the pathogenesis of various autoimmune diseases, making misdiagnosis or delayed treatment common.^[6]^ In this study, we report a case of iMCD in a patient with elevated rheumatoid factor (RF). The patient presented with fatigue, low-grade fever, shoulder erythema, and generalized lymphadenopathy. Laboratory tests showed elevated systemic inflammatory markers. Lymph node pathology revealed reactive hyperplasia of lymphoid tissue with significant increase of interfollicular plasma cells. Considering the elevated RF and associated clinical manifestations, RA-related lymphadenopathy was suspected. As steroid treatment was not effective, we performed a repeat pathological examination, which confirmed the diagnosis of iMCD. Through this study, we aim to provide insights into cases of unexplained lymphadenopathy and offer valuable perspectives in differentiating iMCD from lymphadenopathy caused by other autoimmune diseases.

## 2. Case presentation

**Figure 1.**
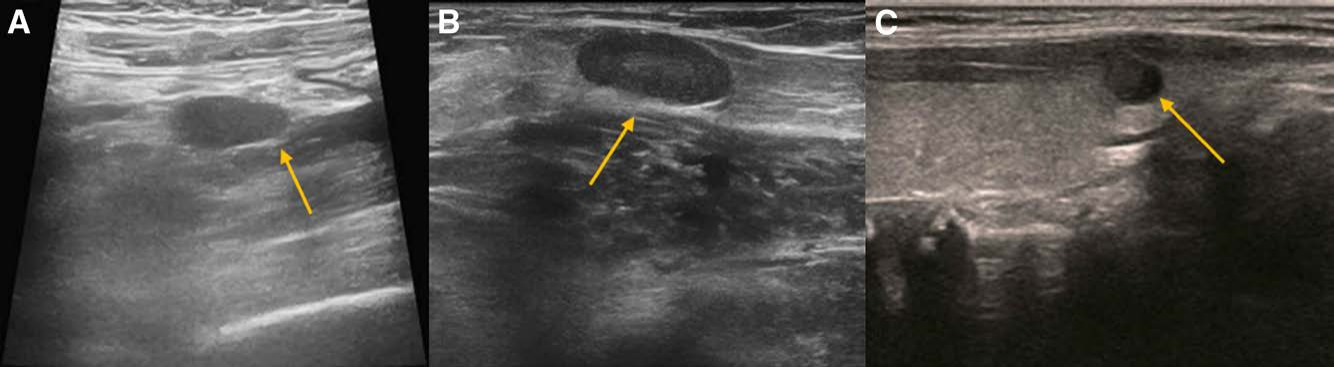
(A) Ultrasound of the retroperitoneal lymph nodes shows enlargement of the left iliac vessels-adjacent lymph node, approximately 2.0 × 0.9 cm (yellow arrow). (B) Ultrasound of the inguinal lymph nodes shows enlargement of multiple lymph nodes, with the largest on the right, approximately 1.2 × 0.6 cm (yellow arrow). (C) Ultrasound of the cervical and axillary lymph nodes shows enlargement of multiple lymph nodes, with the largest on the right, approximately 2.3 × 0.9 cm (yellow arrow).

**Figure 2.**
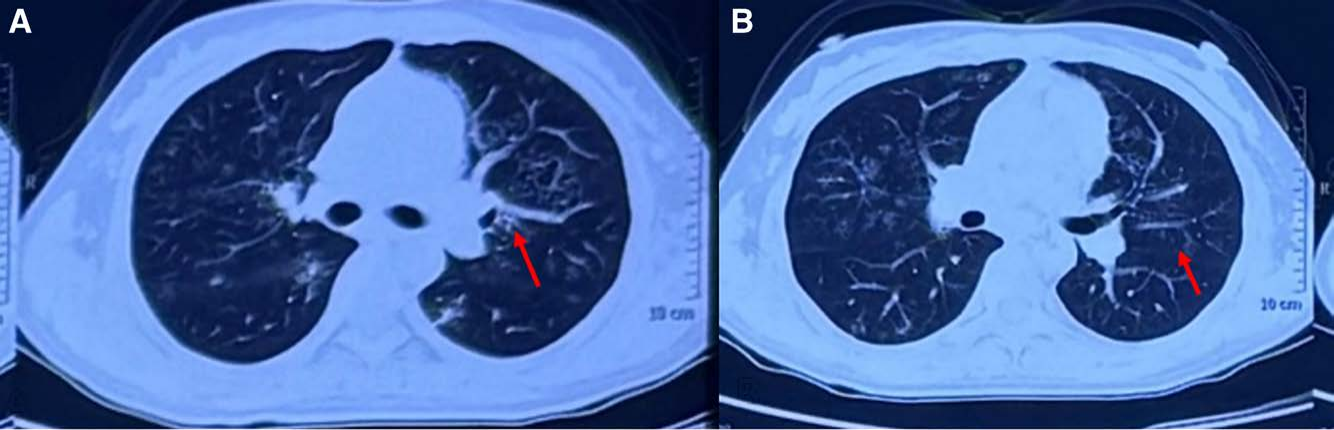
Chest computed tomography (CT) shows diffuse lesions in both lungs. (A) Cystic with accompanying nodules (red arrows); (B) ground-glass opacities (red arrows).

**Figure 3.**
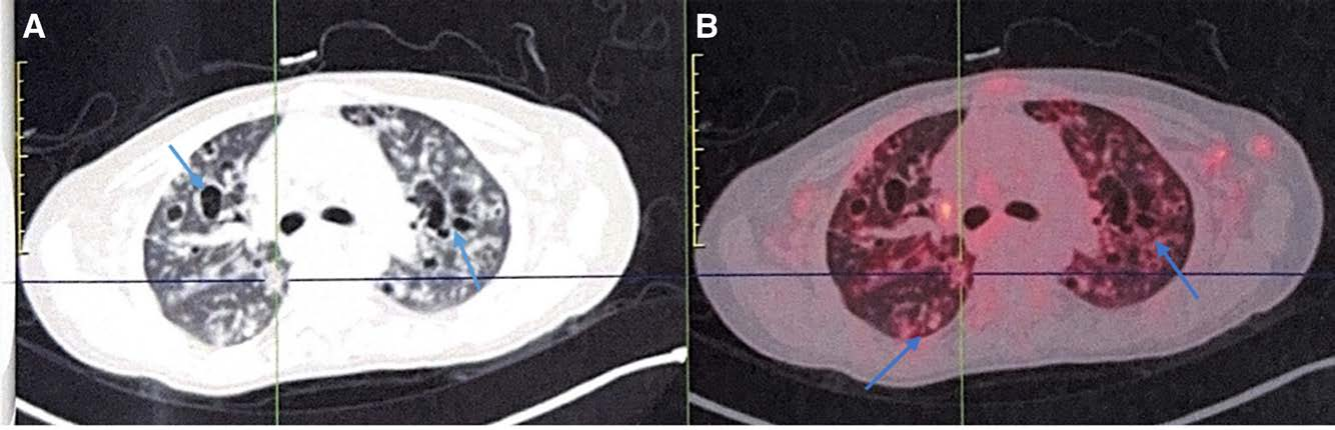
Examination of 18F-fluorodeoxyglucose/positron emission tomography (18F-FDG/PET-CT). (A) Multiple pulmonary nodules (blue arrow); (B) varying degrees of increased FDG metabolism.

**Figure 4.**
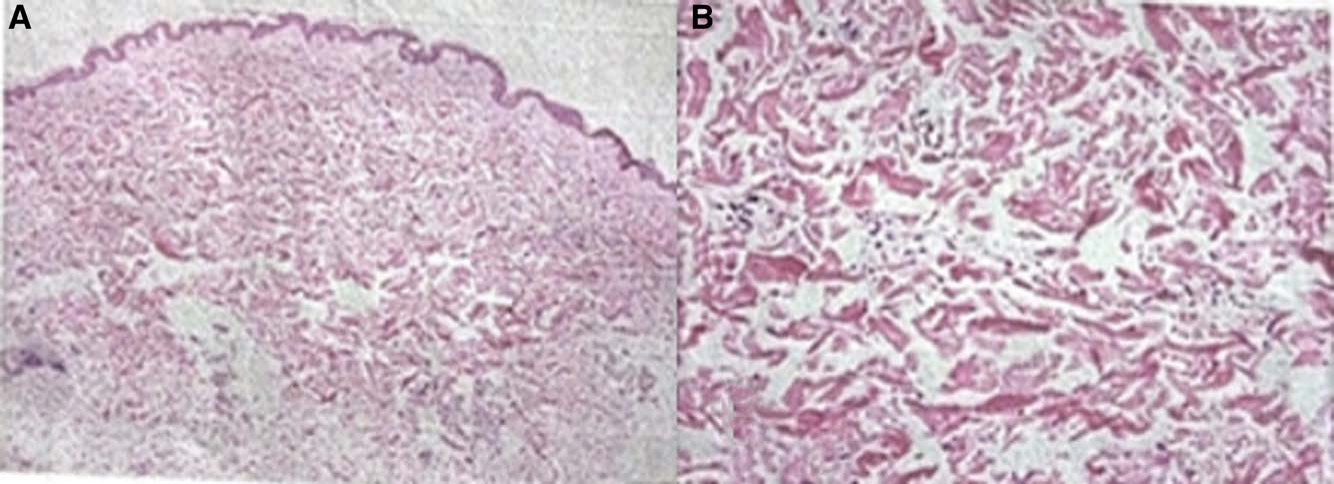
(A) Skin erythema biopsy specimen shows mild epidermal hyperplasia and slight lymphocytic infiltration around superficial to mid-dermal blood vessels in the left shoulder (HE staining, original magnification × 200). (B) Immunohistochemical staining shows CD3 (T cells+), CD20 (B cells‐), CD138 (few scattered+), IgG (‐), IgG4 (few scattered +) (DAB and HE staining, original magnification × 200). Acid-fast staining (‐). HE = hematoxylin and eosin.

**Figure 5.**
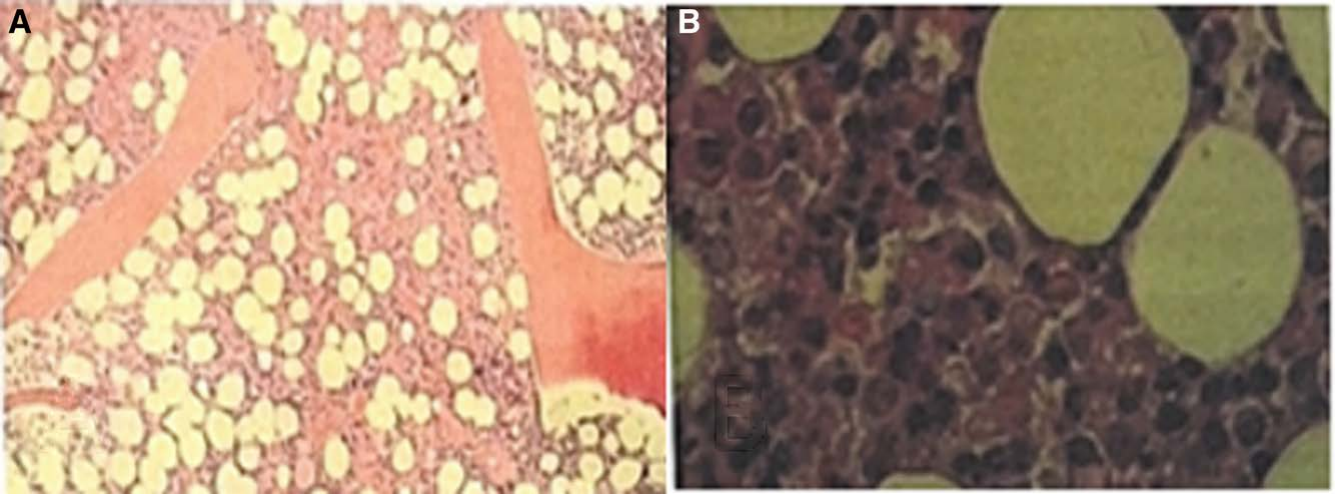
(A) Bone marrow tissue specimens showed low erythroid ratio, marked rouleaux arrangement of erythrocytes; (B) high plasma cell ratio (hematoxylin and eosin [HE] staining, original magnification × 200).

**Figure 6.**
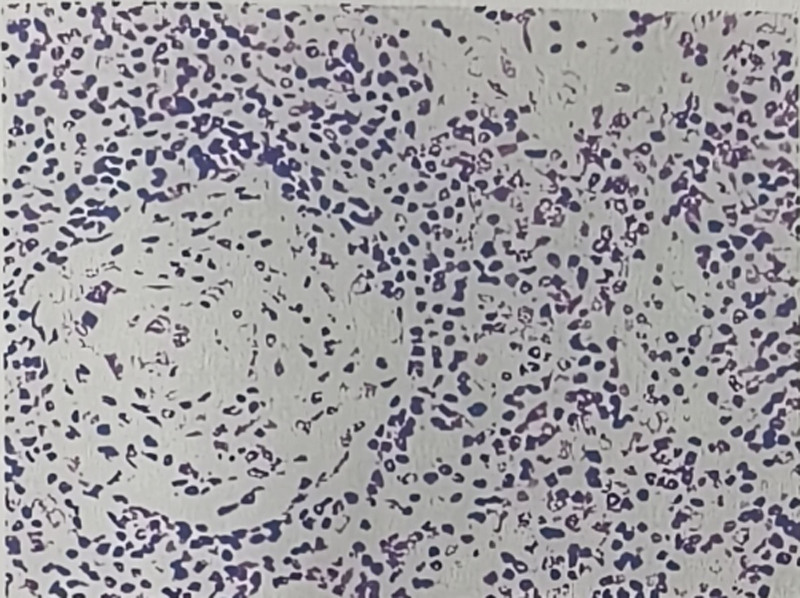
Right axillary lymph node biopsy shows reactive lymphoid hyperplasia (hematoxylin and eosin [HE] staining, original magnification × 200). IgG-4 positive cells are increased, and there is a significant increase in CD38+ and CD138 plasma cells in the interfollicular area (HE staining, original magnification × 200).

This 35-year-old female presented in March 2023 with fatigue and low-grade fever, accompanied by symptoms including shoulder erythema, generalized lymphadenopathy, and a weight loss of 10 kg. Over the past year, the patient had visited several hospitals. Laboratory tests (Table 1) showed persistently decreased levels of hemoglobin and albumin, with sustained elevations in platelet count, serum C-reactive protein, IL-6, and immunoglobulin levels. Elevated RF was observed. No abnormalities were found in serum immunofixation electrophoresis, GM test, Ham test, Coomb test, glucose hemolysis test, sucrose hemolysis test, ANCA, T-SPOT, or gene rearrangement, all of which were negative (Table 1). Multiple lymph nodes in both sides of the neck, supraclavicular fossa, axilla, hilar regions, and mediastinum (Fig. 1);chest computed tomography (CT) showed diffuse bilateral lung lesions (mainly cystic, with faint patches and nodules), suggesting infection? Systemic disease involvement (e.g., iMCD) (Fig. 2). Examination with 18F-fluorodeoxyglucose (FDG) positron emission tomography/computed tomography showed increased FDG uptake in multiple regional lymph nodes, multiple nodules in both lungs, the spleen, and various skeletal and bone marrow sites, suggesting the possibility of a lymphoproliferative disorder (e.g., multicentric Castleman disease?) (Fig. 3). The skin erythema biopsy specimen (left shoulder) showed mild epidermal hyperplasia, with sparse lymphocytic infiltration around superficial to mid-dermal blood vessels, extravasation of red blood cells, and histological features suggestive of allergic purpura. Immunohistochemistry: CD3 (T cells+), CD20 (B cells‐), CD138 (scattered positive), IgG (‐), IgG4 (scattered positive). Acid-fast stain (‐) (Fig. 4). Bone marrow biopsy specimen showed a low ratio of erythroid cells, a high ratio of plasma cells, and erythrocytes arranged in a characteristic coin-like pattern (Fig. 5). Right axillary lymph node biopsy showed reactive lymphoid hyperplasia, with an increase in IgG-4 positive cells, and a significant increase in CD38+ and CD138 plasma cells in the interfollicular areas. Combined with the patient’s medical history, this is consistent with rheumatoid (rheumatic) arthritis-related lymphadenopathy (Fig. 6).

**Table 1:** The patient’s background characteristics and laboratory data (pretreatment and posttreatment partial).

Blood count								
WBC	6.27	10^9^/L	PLT	462	10^9^/L			
RBC	2.54	10^9/L	ESR	115	mm/h			
HB	62	g/L						
Blood chemistry								
CRP	130.32	mg/L	Glb	76.2	g/L	GLU	4.97	mmol/L
Na	133	mmol/L	Alb	22.8	g/L	HbA1c	15.07	%
K	3.82	mmol/L	T-Bil	8.89	µmol/L	IgA	4.42	g/L
Cl	100	mmol/L	AST	13	U/L	IgM	2.62	g/L
BUN	3.3	g/L	ALT	12	U/L	IgE	1090	IU/mL
Cr	47.3	umol/L	ALP	243	U/L	IgG	56.9	g/L
UA	144	umol/L	LDH	117	U/L	IgG4	11,100	mg/L
TP	99	g/L	CK	67	U/L	Ferritin	63.9	ng/mL
Immunologic test								
RF	56.74	IU/ml	Anti-SSB Ab	0.05	AI	IL-6	98.5	pg/mL
ANA	1:00		Anti-RNP Ab	0.34	AI			
Anti-Sm Ab	0.19	AI	MPO-ANCA	0.14				
Anti-SSA Ab	0.1	AI	PR3-ANCA	0.1				
Infection								
GRR	–	G test	–	Ham test	–			
T-SPOT.TB	–	CrAg	–	Coomb test	–			
EBV	–	HBV	–					
IFE	–	HIV	–					
Partial data after treatment								
WBC	6.12	10^9^/L		PLT	222	10^9^/L		
RBC	3.85	10^9^/L		ESR	112	mm/h		
HB	115	g/L						
Blood chemistry								
CRP	16.1	mg/L		IgE	925	IU/mL		
TP	95.3	g/L		IgG	19.9	g/L		
Glb	64.5	g/L		IgG4	5860	mg/L		
Alb	30.8	g/L		Ferritin	64.5	ng/mL		
IgA	1.87	g/L		IL-6	30.86	pg/mL		
IgM	3.42	g/L						

Alb = albumin, ALP = alkaline phosphatase, ALT = alanine transaminase, AST = aspartate transaminase, BUN = blood urea nitrogen, CI = chlorine, CK = creatine kinase, Cr = creatinine, CrAg = *Cryptococcus neoformans* antigen, CRP = C-reactive protein, EBV = Epstein-barr virus, ESR = erythrocyte sedimentation rate, Glb = globulin, GLU = glucose, GRR = gene rearrangement, Hb = hemoglobin, HbA1c = glycated haemoglobin, HBV = hepatitis B virus, HIV = human immunodeficiency virus, IFE = immunofixation electrophoresis, IL-6 = interleukin- 6, K = potassium, LDH = lactic dehydrogenase, MPO-ANCA = myeloperoxidase antineutrophil cytoplasmic antibody, Na = sodium, PLT = platelet, PR3-ANCA = proteinase-3 antineutrophil cytoplasmic antibody, RBC = red blood cell, RF = rheumatoid factor, TBil = total bilirubin, TP = total protein, UA = uric acid, WBC = white blood cell.

After receiving steroid and anti-infection therapy, the patient’s clinical symptoms showed no significant improvement, and laboratory abnormalities persisted. In October 2024, the patient visited our hospital and raised concerns regarding the final lymph node biopsy diagnosis. A repeat lymph node biopsy showed reactive lymphoid hyperplasia, with an increase in IgG-4 positive cells, and a significant increase in CD38+ and CD138 plasma cells in the interfollicular areas, with morphology consistent with Castleman disease (plasmacytic type) (Fig. 6). Follow-up showed an elevated IL-6 level. Therefore, in mid-October 2024, we initiated treatment with tocilizumab, thalidomide, and steroids, which alleviated fatigue and fever and gradually improved hemoglobin, albumin, and C-reactive protein levels (Table 1). Informed consent was obtained from the patient for publication of this case report details.

## 3. Discussion

We encountered a rare case of iMCD in which the patient presented with fever, weight loss, accompanied by shoulder erythema and generalized multiple lymphadenopathy. Laboratory tests showed an elevated RF, and right axillary lymph node biopsy revealed reactive lymphoid hyperplasia, an increase in IgG-4 positive cells, and a significant increase in CD38+ and CD138 plasma cells in the interfollicular areas. The initial diagnosis was RA-related lymphadenopathy, and the patient underwent steroid therapy, which did not yield significant improvement. A reevaluation of the biopsy led to the final diagnosis of iMCD. The patient was then treated with tocilizumab, thalidomide, and steroids, resulting in clinical improvement and a reduction in serum inflammatory markers.

Literature has reported cases of autoimmune diseases combined with CD.^[7]^ There are also reports describing cases of iMCD combined with RA, primarily focusing on the lymph node pathology of iMCD.^[8]^ However, it is rare for patients with iMCD to be misdiagnosed as RA-related lymphadenopathy due to clinical presentation and laboratory/pathological biopsy findings, with the final diagnosis being iMCD. Reports have been made on skin nodular lesions caused by RA, but there are no reports on lymphadenopathy caused by RA.^[9]^ There are also no reported tissue pathological features related to RA lymphadenopathy in tissue biopsies. The initial misdiagnosis may have been due to confusion between the lymph node pathology of iMCD and that of RA-related lymphadenopathy. Additionally, we also referred to the study by Otsuka M et al, who used FDG positron emission tomography/computed tomography to assess the systemic condition of iMCD patients, which did not detect synovitis and further excluded the diagnosis of RA.^[8]^ The elevated RF in this patient may be related to the high IL-6 levels associated with iMCD itself. No significant improvement was seen after receiving steroid treatment alone, which further confirmed that the initial diagnosis of RA-related lymphadenopathy was inappropriate. Additionally, the increased number of IgG4-positive cells in the lymph node pathology immunohistochemistry suggests the possibility of IgG4-RD, which requires us to remain vigilant in clinical diagnosis. The typical characteristics of IgG4-related disease are elevated serum IgG4 levels and a large number of IgG4-positive cells. However, iMCD may also present with elevated serum IgG4 levels and an increased number of IgG4-positive cells, even meeting the histological diagnostic criteria for IgG4-RD.^[10,11]^ Both iMCD and IgG4-RD may involve the skin, but their manifestations are different.^[12]^ Skin involvement in iMCD presents as multiple erythematous to brownish plaques or nodules, with histological features of degenerated follicles and interfollicular plasmacytosis in the dermis and subcutaneous tissue, consistent with the plasmacytic type.^[13]^ In contrast, IgG4-RD presents as erythematous plaques or nodules, with histological observation of nodular infiltration of plasma cells, lymphoid cells, and eosinophils in the deep dermis and subcutaneous tissue.^[14,15]^ Fortunately, our patient presented with skin lesions initially, but no IgG4 deposition was detected in the immunohistochemistry.

## 4. Conclusion

In conclusion, the differential diagnosis of iMCD from autoimmune diseases has always been a challenging issue. We encountered a rare case of iMCD, which was initially misdiagnosed as RA-related lymphadenopathy due to an elevated RF. This raised the question of whether RF could cause lymphadenopathy, and if so, what the pathological tissue should look like. The elevated RF in this patient is likely associated with the high IL-6 levels seen in iMCD. Although iMCD may resemble IgG4-RD due to elevated serum IgG4 levels, and distinguishing between these 2 diseases through serological tests and lymph node histology can be challenging, skin biopsy has helped to differentiate between these 2 conditions. It is unreasonable to make a diagnosis solely based on laboratory tests and pathology in an arbitrary manner. Pathologists should improve their slide-reading skills, and clinicians should comprehensively understand the patient’s condition, considering multiple factors to make the correct diagnosis and prevent misdiagnosis, avoiding unnecessary complications.

## Author contributions

**Conceptualization:** Tongguan Li, Mengjiao Yao, Yanfeng Hou.

**Data curation:** Tongguan Li, Mengjiao Yao.

**Formal analysis:** Tongguan Li.

**Writing – original draft:** Tongguan Li.
